# Development of intelligent hybrid controller for torque ripple minimization in electric drive system with adaptive flux estimator: An experimental case study

**DOI:** 10.1371/journal.pone.0312946

**Published:** 2025-03-28

**Authors:** Surya Kant, Mini Sreejeth, Madhusudan Singh, Ambrish Devanshu, Majed A. Alotaibi, Hasmat Malik, Fausto Pedro García Márquez

**Affiliations:** 1 Department of Electronics and Communication Engineering, Graphic Era Hill University, Bhimtal, Uttrakhand, India; 2 Department of Electrical Engineering, Delhi Technological University, Delhi, India; 3 Department of Electrical Engineering, National Institute of Technology, Silchar, Assam, India; 4 Saudi Electricity Company Chair in Power System Reliability and Security, King Saud University, Riyadh, Saudi Arabia,; 5 Department of Electrical Engineering, College of Engineering, King Saud University, Riyadh, Saudi Arabia,; 6 Department of Electrical Power Engineering, Faculty of Electrical Engineering, University Technology, Malaysia (UTM), Johor, Malaysia; 7 Department of Electrical Engineering, Graphic Era (Deemed to be University), Dehradun, Uttrakhand, India; 8 Ingenium Research Group, Universidad Castilla-La Mancha, Ciudad Real, Spain; Graphic Era Deemed to be University, INDIA

## Abstract

In order to ensure optimal performance of permanent magnet synchronous motors (PMSMs) across many technical applications, it is imperative to minimize torque fluctuations and reduce total harmonic distortion (THD) in stator currents. Hence, this study proposes the utilization of an adaptive flux estimator (AFE) in conjunction with an Intelligent Hybrid Controller (IHC) to mitigate the ripples and total harmonic distortion (THD). The IHC system is constructed by integrating PI and fuzzy logic controllers (FLC) in a cascade configuration, alongside a new switching unit that facilitates automatic switching between the two controllers during various operations of the PMSM. AFE estimates accurate flux which is required to achieve ripple free high dynamic performance of the PMSM drive by using a limiter to fix the flux at reference flux value of the drive. The proposed controller with AFE has achieved its originality through the refinement of membership functions located at the center of the universe of discourse (UOD) and the enhancement of the switching function. These improvements have resulted in increased sensitivity in the proximity to the reference speed. The Fuzzy Logic Controller (FLC) demonstrates superior performance when operating in a transient state, whereas the Proportional-Integral (PI) controller of the proposed system exhibits satisfactory performance under steady-state situations. The efficacy of AFE with IHC is substantiated by the simulation and experimental analysis reported in this study. A significant reduction in both total harmonics distortion (THD) and torque ripples are found.

## Introduction

Alternating current (AC) motor drive systems now can use advanced control techniques due to the development of the latest technologies in power semiconductors and microprocessors. In many industrial applications, it is a necessary requirement that the drive system should be more effective and precise in controlling the speed and torque over a wide range of operating speed. PMSMs are frequently employed in elevators, air compressors, and new energy vehicles due to their benefits of high efficiency, high power density, and low maintenance [1–4].

High switching frequency enhances the output current waveform’s quality, but it also dramatically increases power loss and decreases system efficiency. While low switching frequency minimizes power loss and advances system effectiveness it also generates torque and current ripple. Therefore, it is vital to strike a balance between control performance and effective operation.

Numerous traditional and reliable control methodologies have been documented in the literature for PMSMs. The motor performance is influenced by external disturbances and nonlinear changes in motor characteristics. Current vector control is the most often used control strategy for PMSM drive systems. Predictive control [5–7], sliding mode control [8,9], Adaptive control [10] etc. are used to remove the causes that affect nonlinearity of system. The two most popular methods for regulating the position and speed of PMSM drives are direct torque control (DTC) and field-oriented control (FOC) [11]. Although DTC-based drives produce high torque and flux ripples, they have a greater dynamic response. In FOC-based drives the current, speed, and position loops are independently controlled by controllers, and they offer better steady state performance. In the early days PI controllers were used to control current, speed and position in FOC-based PMSM drive but it degrades drive performance due to complexity in tuning the controller parameters (Kp and Ki) in PI controller. FLC has the capability to remove this constraint of PI controllers [12]. There is no mathematical modelling required in designing FLC because it performs on the basis of user defined linguistic rules [13,14]. FLC is more complicated, computationally expensive, and memory intensive than PI controller. While PI controller offers better performance in steady state condition, FLC works greatly in transient condition of the drive [15–19]. So, an IHC can be constructed which adopts the qualities of PI and FLC for improved dynamic behavior of drive. Torque ripples initiated in motor by cogging torque, armature current harmonics, the magnetic field, and so on. It affects the operational life of motor. Torque ripples can be reduced by improving motor design or employing appropriate motor control technologies. Improving the distribution of winding to increase the spatial magnetic field is a typical strategy used in motor design. The use of skewed slot and fractional slot approaches in the design of stators has proven to be effective in reducing cogging torque [20]. Control algorithms optimize the excitation current of the motor, resulting in lessened torque ripples and a smooth torque output. [21–23].

Main contribution of this paper is given as follows:

- In this paper, an AFC with IHC is realized with main objective of reducing the torque ripples of the PMSM.
- The proposed method includes cascading fuzzy logic and PI controllers, as well as a switching unit that can switch FLC in the case of transient operations and the cascaded PI controller in the case of steady state operations.
- The variation in I/O and O/P variables generate the membership functions (MFs) for FLC. The FLC’s design was made novel by tuning the MFs precisely at the center to offer improved sensitivity in the vicinity of rated speed while optimizing the universe of discourse. The drive’s performance can also be improved by fine-tuning the MFs to account for deviations in i/o and o/p variables. Investigation and comparison of the dynamic performance of the PI controller and the PMSM drive employing the IHC are conducted.

This paper is arranged into five sections comprising Introduction, Principle, and modelling of PMSM through Torque Estimation, Proposed Control Scheme of PMSM Drive, simulation and experimental results followed by Conclusion.

## Principle and modelling of PMSM

To provide decoupled control of stator current and rotor flux, the modelling of PMSM is derived in the α-β [24] as below.

### 1. Voltage and current equation of PMSM

The voltage (${\text{v}}_{\text{sα}}$ , ${\text{v}}_{\text{sβ}}$) equations in the α-β axes are represented below:


$$v_{s\alpha }=R_{s}i_{s\alpha }+\frac{d\psi_{s\alpha }}{dt}$$ (1)

$$v_{s\beta }=R_{s}i_{s\beta }+\frac{d\psi_{s\beta }}{dt}$$ (2)

Where ${\text{R}}_{\text{s}}$ is the stator resistance, ${\text{i}}_{\text{sα}}$ and ${\text{i}}_{\text{sβ}}$ are the per phase stator currents in the α-β axes and $\psi_{\text{sα}}$, $\psi_{\text{sβ}}$ are the stator flux in the α-β axes, which is represented as


$$\psi_{s\alpha }=\int \left(v_{s\alpha }-i_{s\alpha }R_{s}\right)dt=\int e_{s\alpha }dt$$ (3)

$$\psi_{s\beta }=\int \left(v_{s\beta }-i_{s\beta }R_{s}\right)dt=\int e_{s\beta }dt$$ (4)

Where $e_{s\alpha }=\left(v_{s\alpha }-i_{s\alpha }R_{s}\right)$ and$e_{s\beta }=\left(v_{s\beta }-i_{s\beta }R_{s}\right)$

The voltage at DC link, ${\text{V}}_{\text{dc}}$ and the condition of the switches of inverter may be used to depict the stator voltages in the α-β axis as:


$$v_{s\alpha }=\frac{V_{dc}}{3}\left(2S_{1}-S_{3}-S_{5}\right)$$ (5)

$$v_{s\beta }=\frac{V_{dc}}{3}\left[\sqrt{3}\left(S_{3}-S_{5}\right)\right]$$ (6)

where ${\text{S}}_{\text{1}}$, ${\text{S}}_{\text{3}}$ and ${\text{S}}_{5}$ denote the locations of the switches on a voltage source inverter (VSI), with a value of 1 for an off state and a value of 0 for a on state. The stator currents are also described as:


$$i_{s\alpha }=i_{a}$$ (7)

$$i_{s\beta }=\frac{1}{\sqrt{3}}\left(i_{b}-i_{c}\right)$$ (8)

### 2. Adaptive flux estimator

**Fig 1:**
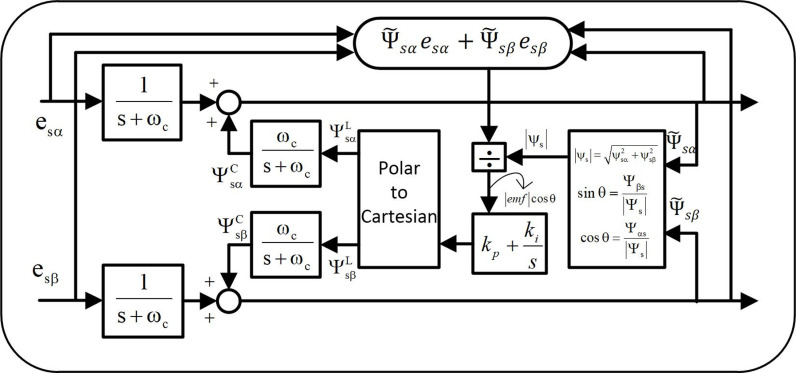
Adaptive flux estimator.

Accurate flux estimation is required to achieve ripple free high dynamic performance of the PMSM drive with different control strategies such as FOC and DTC. Since the inverter is nonlinear, the pure integrator used with the voltage model of the PMSM flux calculation introduces drift signal, sensors problem and many other electronics measuring instruments used while running the machine. Therefore, to overcome this problem a flux estimation technique was proposed in [25,26] for PMSM drives, but it has some limitations with it. In this method [27,28] a limiter was used to fix the flux at reference flux value of AC drive. Therefore, to achieve the variable flux levels an adaptive flux estimator is implemented in this paper as shown in Fig 1.

In this indefinite integral is substituted by a low pass filter added with the compensating feedback signal. Mathematically it can be stated as:


$${\tilde{\psi }}_{s}=e_{s}\frac{1}{s+\omega_{c}}+Z_{comp}\frac{\omega_{c}}{s+\omega_{c}}={\tilde{\psi }}_{s}=\psi_{s}^{1}+\psi_{s}^{2}$$ (9)

Where, $\psi_{s}^{1}=e_{s}\frac{1}{s+\omega_{c}}$ and$\psi_{s}^{2}=Z_{comp}\frac{\omega_{c}}{s+\omega_{c}}$

First part of eqn. (9) is the integrator with back emf, and second part is compensating feedback flux signal to balance the output flux signal. In implemented adaptive flux estimator, motor flux and emf are in phase quadrature of each other. The two components $\Psi_{s\alpha }$ and $\Psi_{s\beta }$ of total flux $\psi_{s}$ comprised with two further components, i.e., feedback and feedforward as shown in Fig 1. Further any variations in these components lead to the phase angle deviations from 90 degrees between the flux and the emf. This deviation leads to the error which is given by the following equations:


$$\left|emf\right|.\cos\phi =\frac{{\tilde{\psi }}_{s\alpha }e_{s\alpha }+{\tilde{\psi }}_{s\beta }e_{s\beta }}{\left|{\tilde{\psi }}_{s}\right|}$$ (10)

Therefore, to eliminate this error, a detector is implemented with PI which is given by the following equation:


$$Z_{comp}=\left(k_{p}+\frac{k_{i}}{s}\right).\frac{{\tilde{\psi }}_{s\alpha }e_{s\alpha }+{\tilde{\psi }}_{s\beta }e_{s\beta }}{\left|{\tilde{\psi }}_{s}\right|}$$ (11)

Implementation of these methods for the operation of PMSM resulting into the lower speed, flux, and torque ripple. If the angle between the estimated flux and the and the emf is 90 degrees, then the output of the PI controller is zero.

### 3. Adaptive torque estimation

The estimated Torque, ${\text{T}}_{\text{est}}$ is represented as given below:


$$T_{est}=\left(\frac{3}{2}\right)\left(\frac{P}{2}\right)\left({\tilde{\psi }}_{s\alpha }i_{s\beta }-{\tilde{\psi }}_{s\beta }i_{s\alpha }\right)$$ (12)

**Fig 2:**
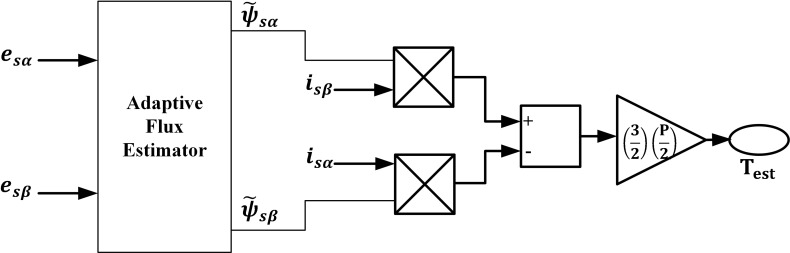
Adaptive torque estimator.

‘P’ stands for the number of poles. Equations 1 to 12 are used to describe the torque estimator suggested here; it is seen in Fig 2.

### 4. PI speed and current controller

The ${\text{e}}_{\text{0}}\text{(k)}$ is output of PI controller and it is expressed as:


$$e_{0}\left(k\right)=K_{p}e\left(k\right)+K_{i}\overset{k}{\underset{0}{\int }}e\left(k\right)dt$$ (13)

Here, $K_{p}$ and ${\text{K}}_{\text{i}}$ are gains of PI, while e(k) is the error signal. By employing a PI controller, the expressions for the speed error${\text{ω}}_{{\text{r}}_{\text{err}}}$ that creates reference torque at the Kth instance, ${\text{T}}_{\text{e}}^{\text{*}}(\text{K})$are given.


$${\text{ω}}_{r_{err}}={\text{ω}}_{r}^{*}-{\text{ω}}_{r}$$ (14)

$$T_{e}^{*}\left(K\right)=\left(K_{p\text{ω}s}+\frac{K_{i\text{ω}s}}{S}\right){\text{ω}}_{r_{err}}\left(K\right)$$ (15)

$$T_{e}^{*}\left(K\right)=\left(K_{p\text{ω}s}+\frac{K_{i\text{ω}s}}{S}\right){\text{ω}}_{r_{err}}\left(K\right)$$ (16)

Where ${\text{ω}}_{r}^{\text{*}}$, ${\text{ω}}_{r}$ and ${\text{ω}}_{r_{err}}\left(K\right)$ are the reference speed, actual speed and speed error at Kth instance respectively; $K_{p\text{ω}s}$ and $K_{i\text{ω}s}$ are gains of PI. The q-axis stator current, ${\text{i}}_{\text{q}}^{\text{*}}(\text{K})$ is produced by the PI controller utilizing the error in torque, ${\text{T}}_{{\text{e}}_{\text{err}}}$ between the command torque, ${\text{T}}_{\text{e}}^{\text{*}}$ and estimated torque, ${\text{T}}_{\text{est}}$.


$$T_{e_{err}}=T_{e}^{*}-T_{est}$$ (17)

$$i_{q}^{*}\left(K\right)=\left(K_{pqs}+\frac{K_{iqs}}{S}\right)T_{e_{err}}\left(K\right)$$ (18)

Where ${\text{K}}_{\text{pqs}}$ and ${\text{K}}_{\text{iqs}}$ are defined as tuning parameter of PI controller respectively and ${\text{T}}_{{\text{e}}_{\text{err}}}(\text{K})\text{ is}$ the torque error at the Kth instance.

### 5. Design of intelligent hybrid controller

**Fig 3:**
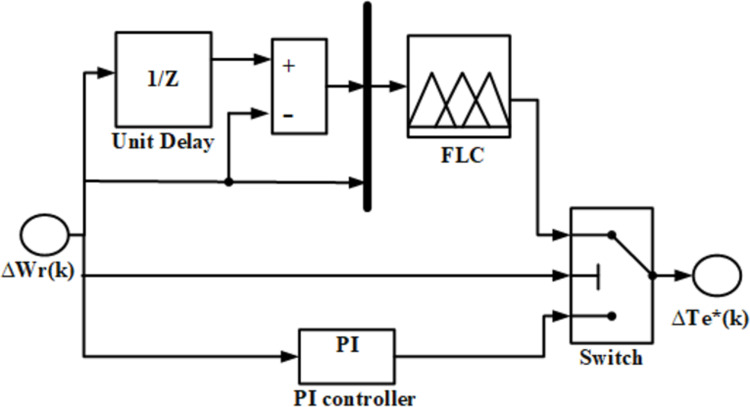
Intelligent hybrid controller.

Soft computing approaches can be used to overcome the performance limits of traditional methods used to control the speed of PMSM drive, that rely on exact models [29,30]. The PMSM drive may experience difficulties with PI controller only to obtain the anticipated dynamic performance and when FLC is employed only then it may not be eliminating steady-state errors due to the absence of integrator. Therefore, a dual approach controller is designed which may overcome the demerits of both PI and FLC controllers. The proposed IHC compares the speed deviation with a certain fixed value. If the deviation is larger than the set value, the FLC works, and the PI controller functions as the set value is greater than the deviation. Fig 3. illustrates the controller proposed in this paper.

The IHC combines the FLC and PI controller through enhanced switching capability to utilize the PI controller during the PMSM drive’s steady state and the FLC during the transient phase. It will depend on oscillations observed in the system that how will switch operate. Below is a description of the FLC algorithm created for PMSM drive speed control.

1. Determine I/O parameters.
2. Select MFs and state control rules.
3. State probable inference with control rules and MFs.
4. Transform the fuzzy into crisp set.
5. To get the required performance, adjust the input and output gains properly.

Here input crisp variables are speed error, $\Delta {\text{ω}}_{r}\left(k\right)$ and change in speed error, $\Delta e\left(k\right)$ which are described as:


$$\Delta {\text{ω}}_{r}\left(k\right)=\omega_{r}^{*}\left(k\right)-\omega_{r}\left(k\right)$$ (19)

$$\Delta e\left(k\right)=\Delta {\text{ω}}_{r}\left(k\right)-\Delta {\text{ω}}_{r}\left(k-1\right)$$ (20)

**Fig 4:**
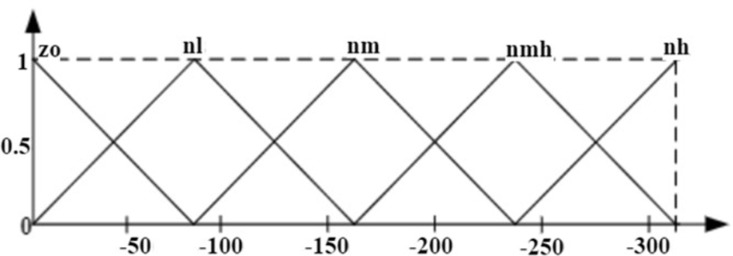
MFs of$\Delta e\left(k\right)$.

**Fig 5:**
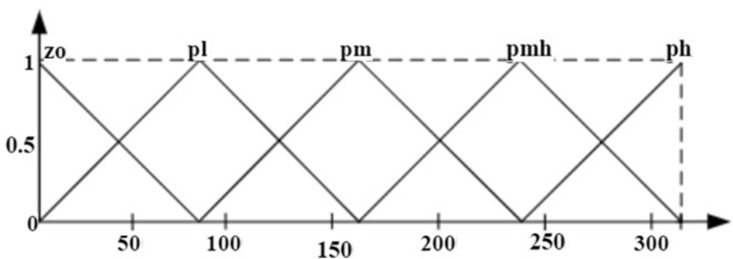
MFs of$\Delta {\text{ω}}_{r}\left(k\right)$.

Output is torque component, which is stated as


$$\Delta T_{e}^{*}\left(k\right)=\Delta T_{e}^{*}\left(k-1\right)+µ.\Delta T_{e}^{*}\left(k\right)$$ (21)

where $\Delta T_{e}^{\text{*}}\left(k\right)$ is commanded value of torque at the $k^{th}$ sample time and µ  is the gain. Fuzzyfication, rule execution, and defuzzyfication are the three main stages that make up the FLC’s operation. Triangular membership functions (MFs) of I/O convert crisp variables into fuzzy variables in the first stage. The MFs for the two inputs, $\Delta e\left(k\right)$, $\Delta {\text{ω}}_{r}\left(k\right)$ are shown in Fig 4 and Fig 5. For I/O variables, the IHC defines fifteen linguistic variables. The first input, e(k), has five linguistic variables: ***zo, nl, nm, nmh, nh.*** Likewise, the second input has ***zo, pl, pm, pmh, ph***. Thus, ***zo, pl, pm, pmh, and ph*** are the five output linguistic variables. The ***zo, nl, nm, nmh, nh, pl, pm, pmh, ph*** represents zero, negative low, negative medium, negative medium high, negative high, positive low, positive medium, positive medium high and positive high respectively.

Using fuzzy variables derived from expert knowledge, a set of control rules is constructed and put into action in the second stage. Fuzzy rules are stated as

IF $\Delta \omega_{r}\left(k\right)$ is ‘a’, $\Delta e\left(k\right)$ is ‘b’ THEN $\Delta T_{e}^{*}\left(k\right)$ is ‘c’.

Here a, b and c are defined as fuzzy sets.

To produce computable results in fuzzy logic, fuzzy sets, and membership functions, fuzzy variables are transformed into crisp variables in the third phase, known as defuzzyfication.. Table 1 lists the fuzzy rules utilized in the IHC.

**Table 1:** Fuzzy rules of IHC.

$\Delta T_{e}^{*}\left(k\right)$		$\Delta e\left(k\right)$				
		*zo*	*nl*	*nm*	*nmh*	*nh*
$\Delta {\text{ω}}_{r}\left(k\right)$	*zo*	*zo*	*pl*	*pmh*	*pmh*	*ph*
	*pl*	*pl*	*zo*	*pl*	*pmh*	*ph*
	*pm*	*pmh*	*pl*	*zo*	*pl*	*pmh*
	*pmh*	*pm*	*pmh*	*pl*	*pl*	*pl*
	*ph*	*ph*	*pm*	*pmh*	*pl*	*zo*

## Proposed control scheme of PMSM drive

**Fig 6:**
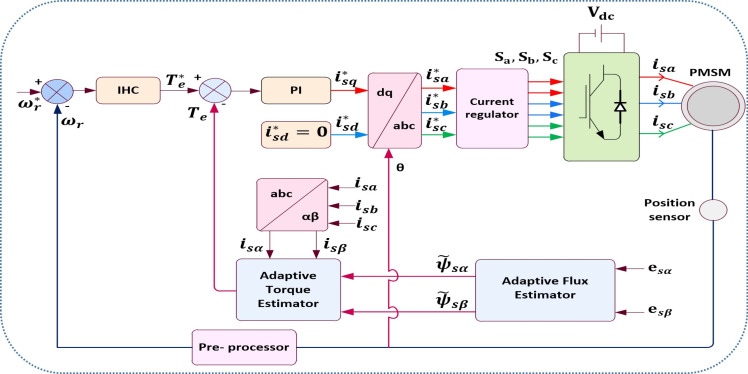
PMSM Drive with IHC.

To regulate the space vectors of voltage, current, and flux, vector control uses a field-oriented technique [31,32]. High bandwidth inner loops are necessary in high performance drives in order to properly measure current, shorten the transient time, and make sure that VSI may be utilized as an amplifier [33–36]. Fig 6. demonstrates PMSM drive system with IHC.

The reference torque command is created by the IHC in the outer speed loop and contrasted with the estimated torque. The voltage at DC link, 3-phase currents ($i_{abc}$) and the switches positions (S1, S3 and S5) of VSI from the pulses generated by the current regulator as stated before are used to determine the torque. The error signal obtained by comparing the estimated torque to the reference torque is given to the current controller, which produces $i_{q}^{\text{*}}$.

The 3-phase reference currents of stator,$i_{a}^{\text{*}}$, $i_{b}^{\text{*}}$ and $i_{c}^{\text{*}}$are computed via Parks Transformation utilizing the rotor position from the encoder (*θ*), and quadrature and direct axis current,$i_{q}^{\text{*}}$ & $i_{d}^{\text{*}}$.


$$i_{a}^{*}=i_{q}^{*}\cos\theta +i_{d}^{*}\sin\theta$$ (22)

$$i_{b}^{*}=i_{q}^{*}\cos\left(\theta -\frac{2\pi }{3}\right)+i_{d}^{*}\sin\left(\theta -\frac{2\pi }{3}\right)$$ (23)

$$i_{c}^{*}=i_{q}^{*}\cos\left(\theta +\frac{2\pi }{3}\right)+i_{d}^{*}\sin\left(\theta +\frac{2\pi }{3}\right)$$ (24)

Where $i_{d}^{\text{*}}=0$ upto rated speed operation. Switching pulses are generated by comparing these reference currents with the triangular wave and fed to the inverter.

## Emulated results demonstration

In this section a simulation model of a 3.4-kW PMSM with the parameters listed in Table 2 is built in MATLAB/Simulink. The simulations are carried out to compare the proposed approach to the traditional PI control scheme and verify it at the sample time of 10µs.

**Table 2:** Parameters of PMSM.

S.No.	Parameters	Rating
1	Power,$P_{in}$	3.4kW
2	Voltage, V	380V
3	Current, I	11 Nm
4	Torque, T	6.9 A
5	Speed, N	314r/sec
6	Poles, P	8
7	Resistance,$R_{s}$	1.93 Ω
8	Inductance,$L_{s}$	0.0114 H
9	Motor Inertia, J	0.11 Kg-M^2^

### 1. Rated speed operation of PMSM drive

**Fig 7:**
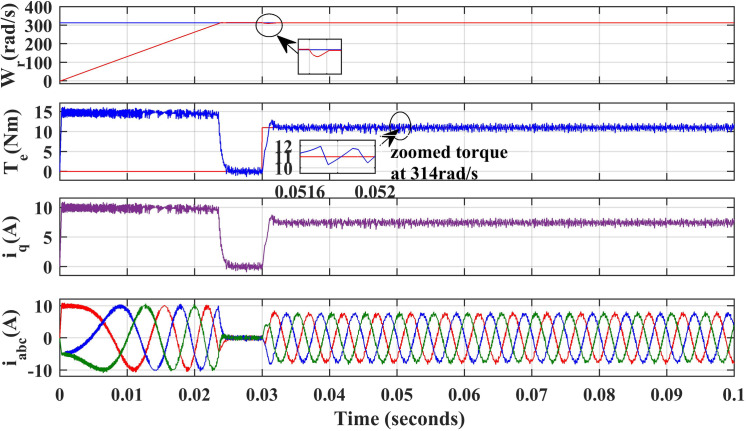
Simulation waveforms of $W_{r}$, $T_{e}$, $i_{q}$ and $i_{abc}$ of PMSM Drive at 314r/sec for using PI.

**Fig 8:**
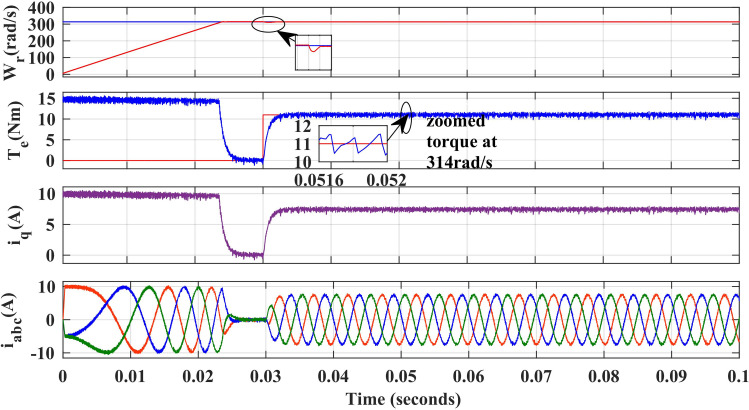
Simulation waveforms of $W_{r}$, $T_{e}$, $i_{q}$ and $i_{abc}$ of PMSM Drive at 314r/sec using IHC.

Figs 7 and 8 illustrate the simulated results of the PMSM drive’s speed, torque, and stator currents when operating at 314 rad/sec, the rated speed with PI and IHC. To verify effectiveness of IHC the motor starts to rotate at speed of 314r/sec under zero load. A sudden fall is observed in speed response for very less time as load torque of 11Nm is applied at 0.03 seconds after that the drive recovers the reference speed again. It can be seen from the torque waveform in Fig 7 and Fig 8 that the IHC controller is more successful in reducing torque ripples than PI controller.

### 2. Drive performance with speed variation

**Fig 9:**
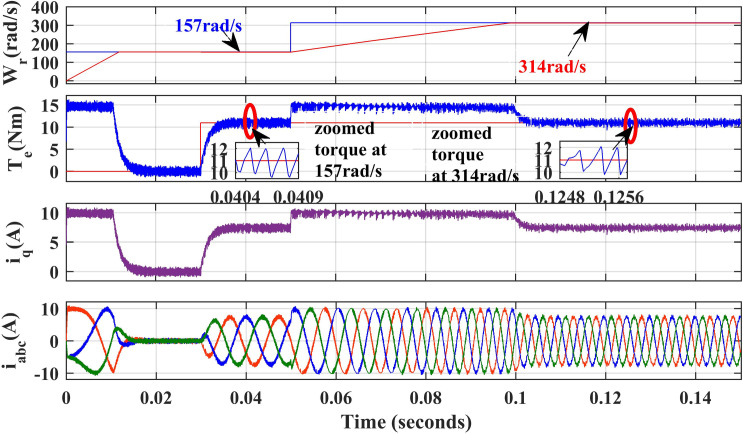
Simulation waveforms of $W_{r}$, $T_{e}$, $i_{q}$ and $i_{abc}$of PMSM Drive at variable speed using PI.

**Fig 10:**
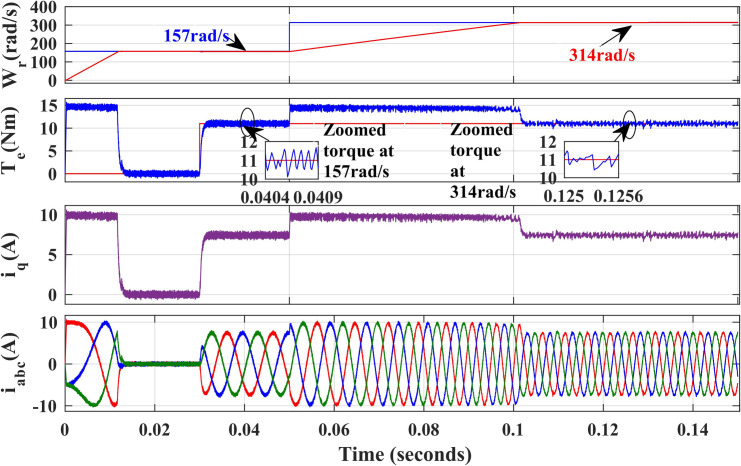
Simulation waveforms of $W_{r}$, $T_{e}$, $i_{q}$ and $i_{abc}$ of PMSM Drive at variable speed using IHC.

Figs 9 and 10, illustrate the simulated results of both PI and IHC controller employed in PMSM drive at speed varying from 157r/sec to 314r/sec. At 157 r/sec, the motor is started at zero load torque. At 0.05s, step variation of speed is given. Initially the motor is running at no load and at 0.03 sec, 11Nm load is inserted. From the waveform given in Fig 9 and Fig 10, it is important to note that the suggested IHC outperforms the traditional PI in both steady-state and dynamic torque performance, with significantly reduction in ripples.

### 3. Drive performance in reverse rotation mode

**Fig 11:**
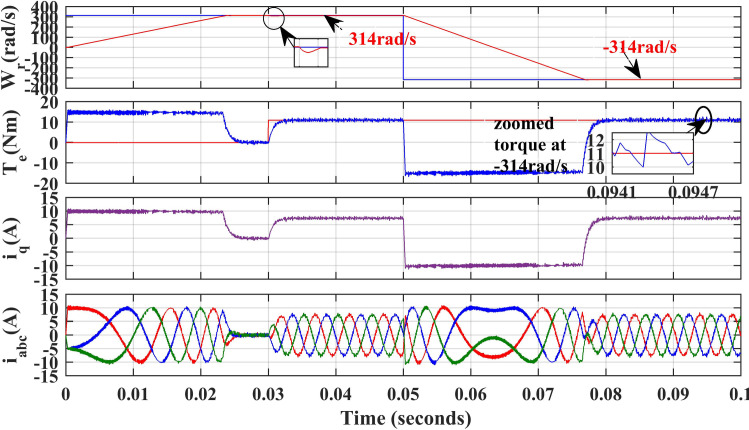
Simulation waveforms of $W_{r}$, $T_{e}$, $i_{q}$ and $i_{abc}$ of PMSM Drive in reverse rotation of speed using PI.

**Fig 12:**
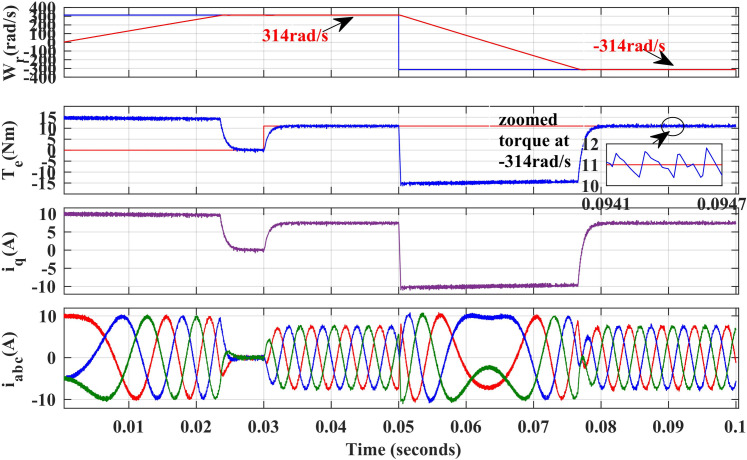
Simulation waveforms of $W_{r}$, $T_{e}$, $i_{q}$ and $i_{abc}$of PMSM Drive at reverse rotation speed using IHC.

Figs 11 and 12, illustrate the simulated results of both PI and IHC controller employed in PMSM drive with AFC at speed varying from 314r/sec to -314r/sec. Initially reference motor speed of rated value is given and at 0.05 sec step variation in speed 314r/sec to -314r/sec in reverse direction is applied to the motor. The motor speed and torque are observed to seamlessly track their set values. According to the torque waveform given in Fig 11 and Fig 12, there is a significant difference in torque ripples induced by both control methods. It can therefore be stated that proposed method can reduce the ripples in motor torque effectively over a conventional method.

### 4. Steady state characteristics of estimated torque and measured torque

**Fig 13:**
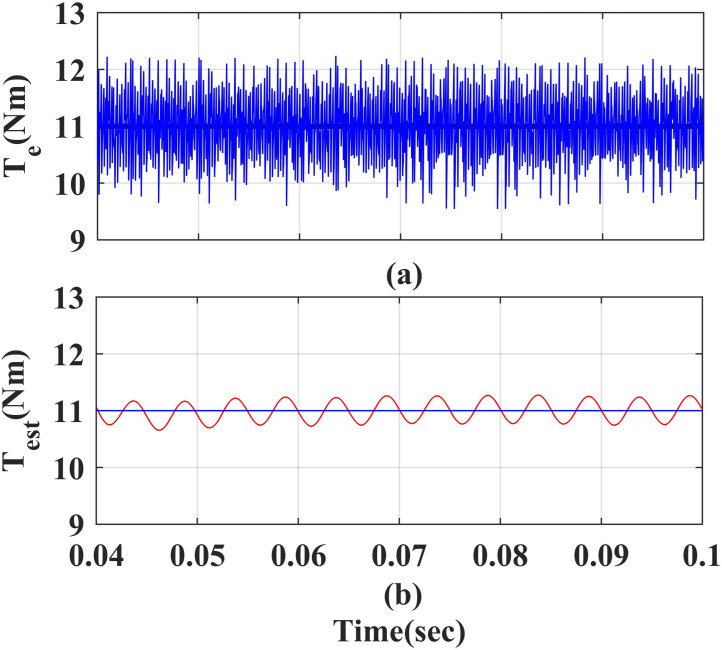
Measured and estimated values for the torque of the PMSM using the PI.

**Fig 14:**
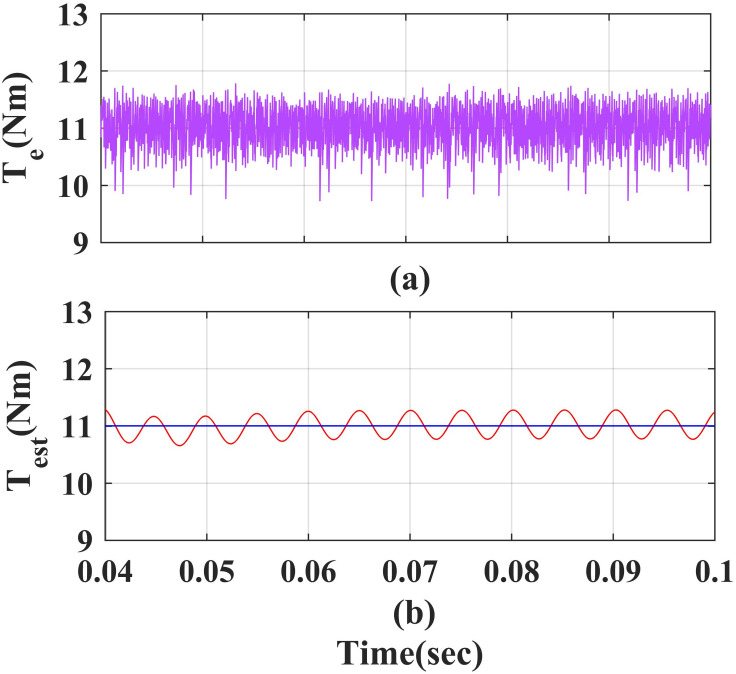
Measured and estimated values for the torque of the PMSM using the IHC.

Fig 13 and Fig 14 show steady state measured value and estimated value of torque of the PMSM drive using PI and IHC with AFC. The measured value of torque (T_e_) is 11Nm and estimate value of torque (T_est_) is 10.9 Nm at rated speed. The average error between both torque values is observed to be 0.1Nm.

### 5. Calculation of torque ripple

Torque ripple is calculated using Equation 25 as given below: 


$$\text{Torque ripple }\left(\text{\%}\right)\text{=}\frac{T_{max}-T_{min}}{T_{e}}\times 100$$ (25)

**Fig 15:**
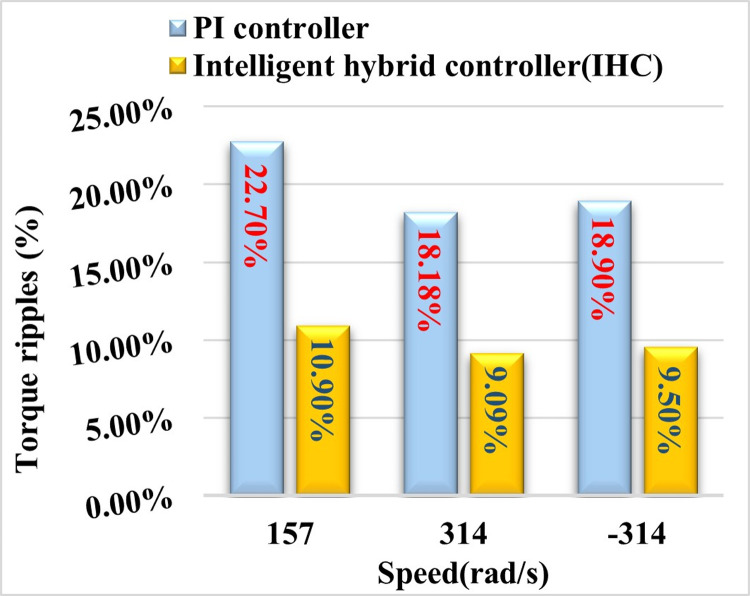
Torque ripples in PMSM with PI & IHC at various speeds.

Here $T_{max}$, $T_{min}$ are maximum and minimum peak of toque which is observed from given torque waveforms. The percentage values of torque ripples running at various speeds with a PI and IHC in PMSM drive is shown in Fig 15. When employing IHC as a speed controller instead of a PI controller, ripples are seen to be minimized.

### 6. THD analysis

**Fig 16:**
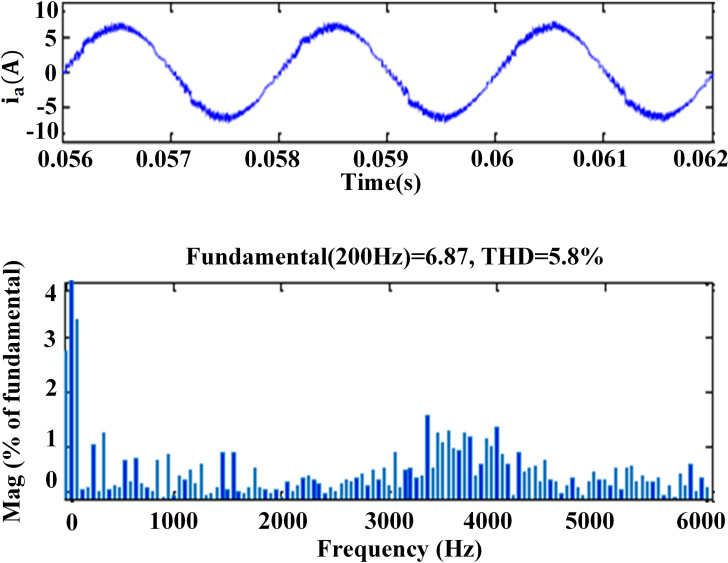
THD in motor current at 314r/sec with PI.

**Fig 17:**
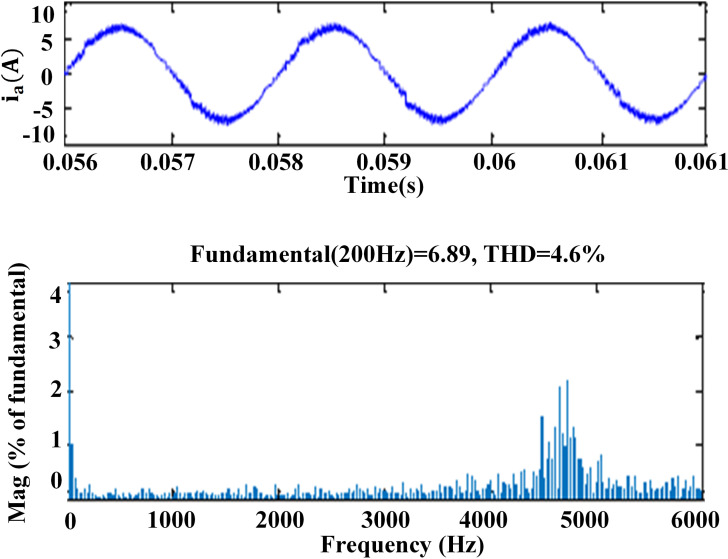
THD in motor current at 314r/sec with IHC.

**Fig 18:**
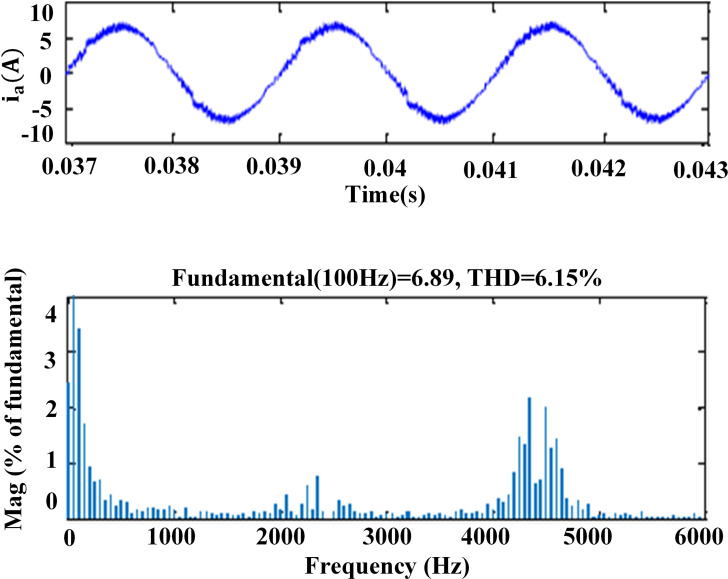
THD in motor current at 157r/sec with PI.

**Fig 19:**
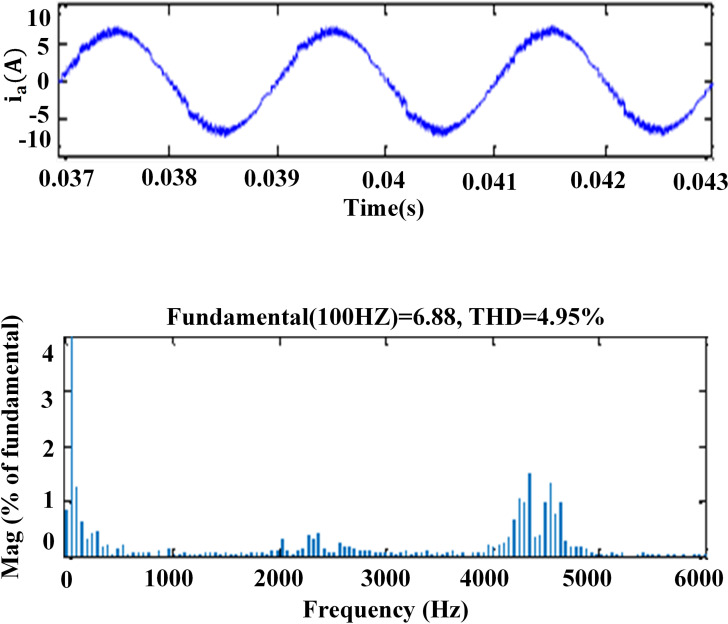
THD in motor current at 157r/sec with IHC.

The harmonic spectra of the stator current of PMSM with PI and IHC at 314r/sec is shown in Fig 16 and Fig 17. THD measured with PI controller is 5.8%, whereas THD measured with IHC is 4.6%. The harmonic spectra of the stator current for the drive with PI and IHC is shown in Fig 18 and Fig 19 while it is operating at half of its rated speed (157 rad/sec). THD is reported to be 4.95% with IHC and 6.15% with PI controller. The average value of switching frequency for all techniques is fixed constant at 7.5 kHz for simulation experiments. When THD is examined up to 6 kHz, it is found that the PMSM drive with Intelligent Hybrid Controller has lower THD in stator current than the PI controller.

## Experimental results demonstration

**Fig 20:**
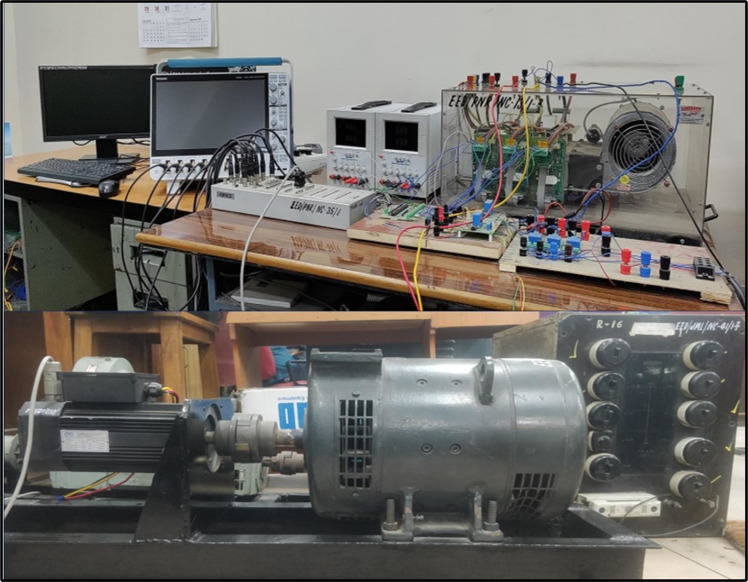
Experimental bench of PMSM drive.

An experimental setup is developed for the operation of PMSM drive shown in Fig 20. The ds1104 controller, VSI, current and voltage, driver gating circuit, and power sources are the basic components used in the experimental setup of PMSM drive. MATLAB/Simulink and dSPACE1104 are used to implement the different controllers for PMSM drives. Table 1 lists the PMSM parameters utilized for the experimental investigation. The VSI is controlled by dS1104 controller and supplies the stator voltage for the PMSM. A DC generator is paired on the same shaft to provide electrical loading for the PMSM.

### 1. Experimental performance of PMSM drive

**Fig 21:**
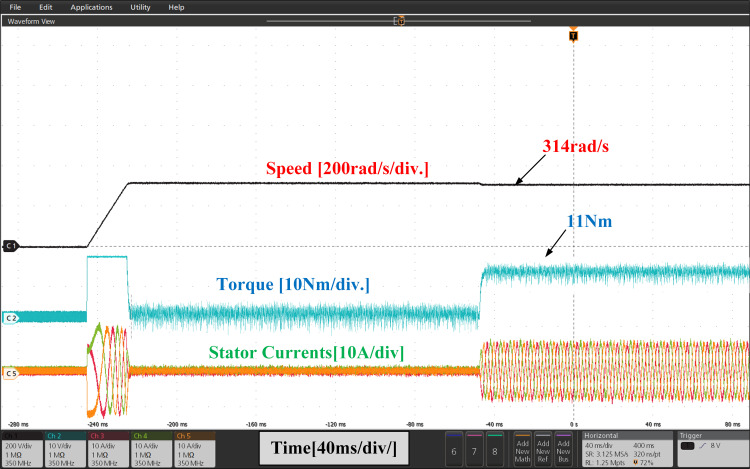
Experimental waveform of $W_{r}$, $T_{e}$ and $i_{abc}$ of motor running at 314r/sec using PI.

**Fig 22:**
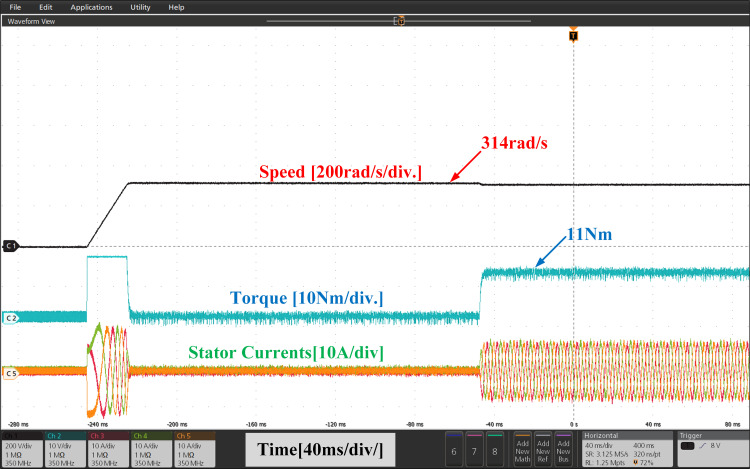
Experimental waveform of $W_{r}$, $T_{e}$ and $i_{abc}$ of motor running at 314r/sec using IHC.

Fig 21, and Fig 22, show the experimental results of both PI and IHC employed in PMSM drive operated at 314r/sec. In starting the motor starts to rotate with full speed at zero load and easily trajectory the speed given in reference. When load of 11Nm is inserted at the shaft, a quick drop is perceived in speed curve and after few second the drive trajectories the reference speed effortlessly again. It can be seen from the torque waveform in Figs 7 and 8 that the IHC controller is more successful in reducing torque ripples than PI controller.

**Fig 23:**
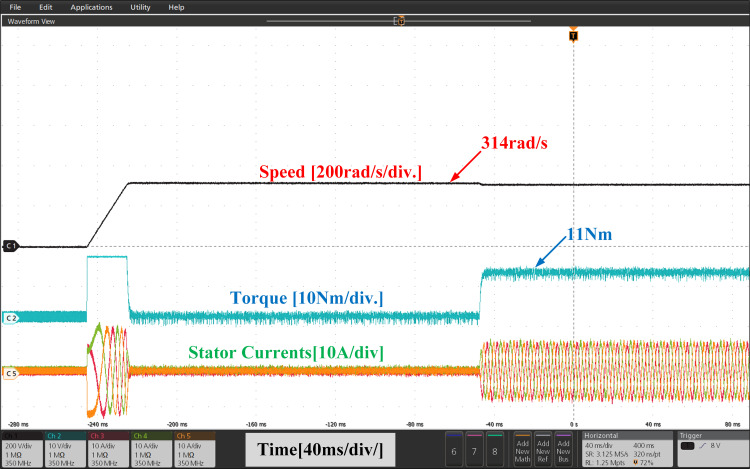
Experimental waveform of $W_{r}$,$T_{e}$ and $i_{abc}$ of motor with linear increase in speed using PI.

**Fig 24:**
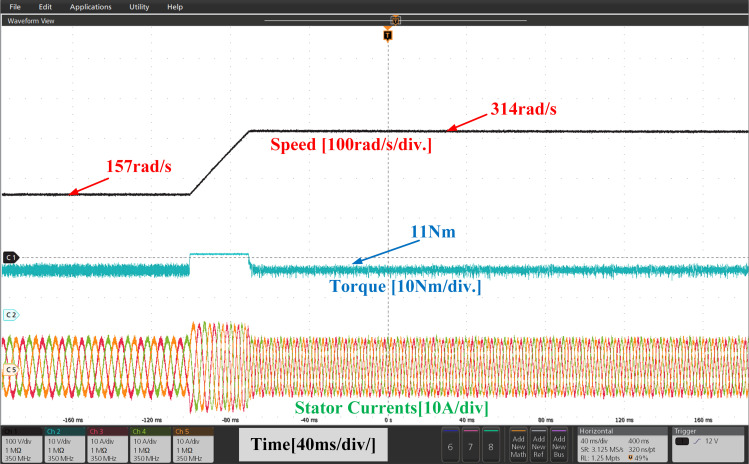
Experimental waveform of $W_{r}$, $T_{e}$ and $i_{abc}$ of motor with linear increase in speed using IHC.

Fig 23 and Fig 24, illustrate the experimental results of both PI and IHC controller employed in PMSM drive at speed varying from 157r/sec to 314r/sec. At 157 r/sec, the motor is started at zero load torque. The motor is started to run at zero load condition at 157r/sec. A constant load of 11Nm is inserted to the shaft and it is perceived that the motor generates the net demanded torque without any interval. After motor reaches in steady state, a change in commanded speed from 157r/sec to 314r/sec is applied to motor. A rise in load torque is observed during the speed variation and it reaches again at rated value. The motor speed and torque are found to follow their set values. The IHC greatly reduces toque ripples, according to experimental analysis of the torque response of motor.

**Fig 25:**
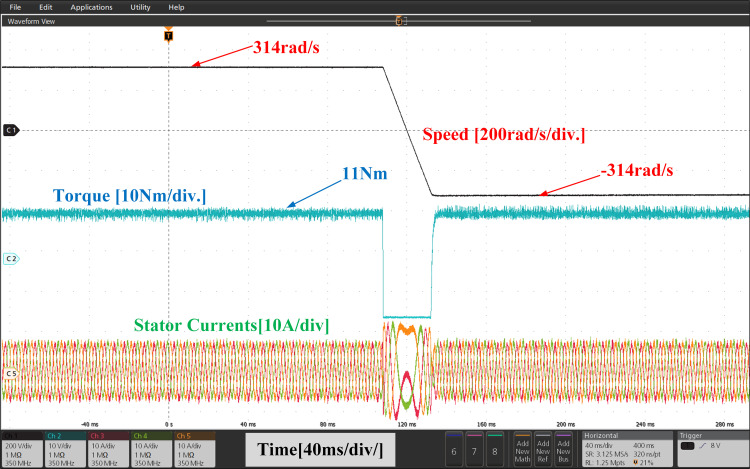
Experimental waveform of $W_{r}$, $T_{e}$ and $i_{abc}$ of motor with reverse rotation of speed using PI.

**Fig 26:**
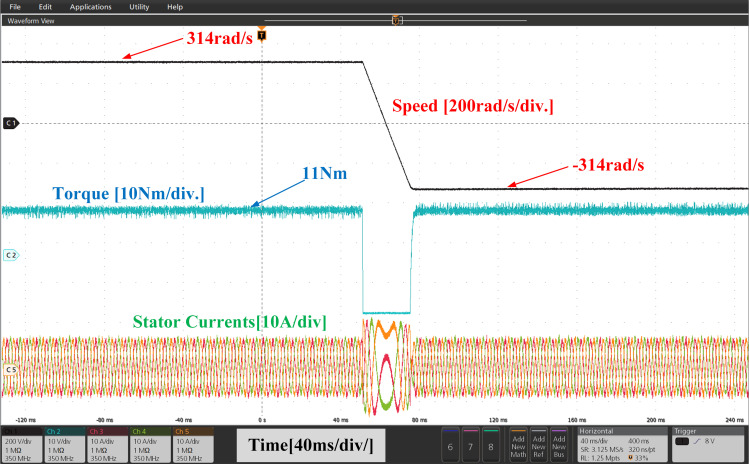
Experimental waveform of $W_{r}$, $T_{e}$ and $i_{abc}$ of motor with reverse rotation of speed using IHC.

Fig. 25 and Fig. 26, illustrate the experimental results of both PI and IHC controller employed in PMSM drive with AFC at speed varying from 314r/sec to -314r/sec. Initially reference motor speed of rated value is given and after reaching at steady state condition a step variation in speed 314r/sec to -314r/sec in reverse direction is given to the motor. A drop in motor torque is witnessed during change in speed and it reaches again at rated value after few seconds. The motor speed and torque are observed to seamlessly track their set values. According to the torque waveform given in Figs 25 and 26, there is a significant difference in torque ripples induced by both control methods. It can therefore be stated that proposed method can reduce the ripples in motor torque effectively over a conventional method.

### 2. THD analysis

**Fig 27:**
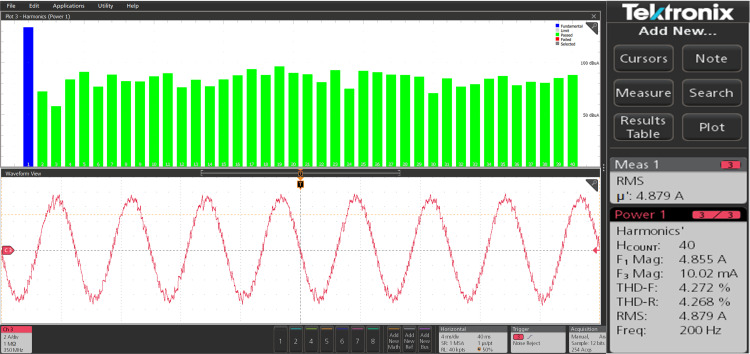
Current THD of PMSM at 314 r/sec with PI.

**Fig 28:**
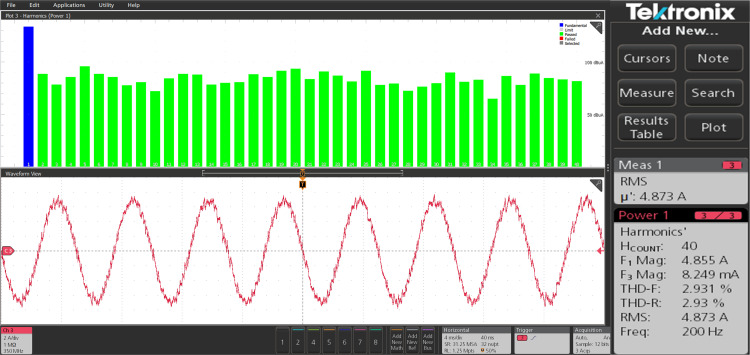
Current THD of PMSM at 314 r/sec with IHC.

**Fig 29:**
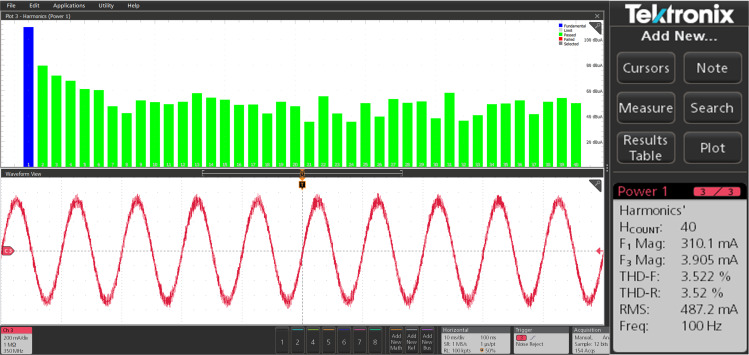
Current THD of PMSM at 157 r/sec with PI.

**Fig 30:**
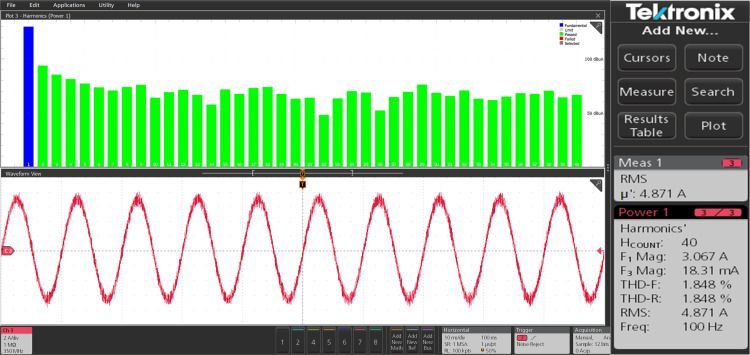
Current THD of PMSM at 157 r/sec with IHC.

The harmonic spectra of the stator current of PMSM with PI and IHC at rated speed of 314r/sec is shown in Figs 27 and 28. THD measured with PI controller is 4.272%, whereas THD measured with IHC is 2.931%. The harmonic spectra of the stator current for the drive with PI and IHC is shown in Figs 29 and 30 while it is operating at half of its rated speed (157 rad/sec). THD is reported to be 1.848% with IHC and 3.522% with PI controller. When THD is examined by performing experiment, it is found that the PMSM drive with Intelligent Hybrid Controller has lower THD in stator current than the PI controller.

## Conclusion

This study proposes an intelligent hybrid controller for Permanent Magnet Synchronous Motor (PMSM) drives, aiming to mitigate the problems arising from the independent operation of standard Proportional-Integral (PI) controller and Fuzzy Logic Controller (FLC). The proposed controller incorporates an adaptive flux and torque estimator. The controller under consideration is formulated by integrating a Proportional-Integral (PI) controller and a Fuzzy Logic Controller (FLC) in a cascaded manner, incorporating innovative switching functionalities. A unique methodology is proposed for the estimation of flux, with the aim of reducing disturbances in current, torque, and flux. In addition, comprehensive simulations and experimental findings have provided evidence that the suggested control methodology presents advantages such as decreased fluctuations in motor torque, lower total harmonic distortions in current, and enhanced overall drive performance in both steady-state and transient states across various operational scenarios, in contrast to the conventional proportional-integral (PI) controller. The torque ripples in motor are observed as 27.70%, 18.18% and 18.90% with PI controller and 10.90%, 9.09% and 9.50% with IHC controller at speed of 157 rad/s, 314 rad/s and -314 rad/s respectively. It shows that IHC can give better performance with reduced ripples in torque than PI controller. THD measured with PI controller is 5.8%, whereas THD measured with IHC is 4.6% at speed of 314 rad/s and 6.15% with PI controller, 4.95% with IHC at speed of 157 rad/s. Thus, IHC controller provides lesser THD in current than PI controller at different operating speed. In future work, the proposed method can be implemented for the electric vehicle application with some adaptive controller based on artificial intelligence.

## References

[1] Harib KH, Khousa EA, Ismail A. Field oriented motion control of a 3 phase permanent magnet synchronous motor. In 2nd International Conference on Electric Power and Energy Conversion Systems (EPECS). Sharjah; 2011. p. 1–7.

[2] Rahman MA, George GH, Radwan TS, Uddin MN. Performance of current controllers for VSI-fed IPMSM drive. IEEE Trans Ind Applicat. 2000;36(6):1531–8. doi: 10.1109/28.887203

[3] Pillay P, Krishnan R. Modeling, simulation, and analysis of permanent-magnet motor drives. part I. The permanent-magnet synchronous motor drive. IEEE Trans Ind Applicat. 1989;25(2):265–73. doi: 10.1109/28.25541

[4] Dhaoudi R, Mohan N. Analysis of current regulated voltage source inverter for permanent magnet synchronous motor drives in normal and extended speed ranges. IEEE Trans Energy Convers. 1990;5(1):137–44.

[5] Alireza AK, Davood R, Jose R. Predictive control of permanent magnet synchronous motor with non-sinusoidal flux distribution for torque ripple minimization using the recursive least square identification method. IET Electric Power Applicat. 2017;11(5):847–56.

[6] Türker U, Buyukkeles AFB. A robust predictive current controller for PMSM drives. IEEE Trans Ind Electron. 2016;63(6):3906–14.

[7] Suryakant M, Sreejeth M, Singh AK. Minimization of torque ripples in PMSM drive using PI- resonant controller-based model predictive control. ElectrEng. 2022;105:207–19.

[8] Qiao Z, Shi T, Wang Y, Yan Y, Xia C, He X. New sliding-mode observer for position sensorless control of permanent-magnet synchronous motor. IEEE Trans Ind Electron. 2013;60(2):710–9. doi: 10.1109/tie.2012.2206359

[9] Mani P, Rajan R, Shanmugam L, Joo YH. Adaptive fractional fuzzy integral sliding mode control for PMSM model. IEEE Trans Fuzzy Syst. 2019;27(8):1674–86. doi: 10.1109/tfuzz.2018.2886169

[10] Wai R-J, Chen M-W, Liu Y-K. Design of adaptive control and fuzzy neural network control for single-stage boost inverter. IEEE Trans Ind Electron. 2015;62(9):5434–45. doi: 10.1109/tie.2015.2408571

[11] Merzoug MS, Naceri F. Comparison of field-oriented control and direct torque control for permanent magnet synchronous motor. Int J Elect Comput Eng. 2008;2(9):1797–801.

[12] Asgharpour-alamdari H, Alinejad-beromi YH, Yaghobi Y. A fuzzy-based speed controller for improvement of induction motor’s drive performance. Iran J Fuzzy Syst. 2016;13(2):61–70.

[13] Uddin MN, Rahman MA. Fuzzy logic based speed control of an IPM synchronous motor drive. Engineering Solutions for the Next Millennium. IEEE Canadian Conference on Electrical and Computer Engineering. Alberta, Canada: 1999. p. 1259–64.

[14] Uddin MN, Chy MMI. A novel fuzzy-logic-controller-based torque and flux controls of IPM synchronous motor. IEEE Trans Ind Applicat. 2010;46:1220–9.

[15] Sant A, Rajagopal KR. PM synchronous motor speed control using hybrid fuzzy-PI with novel switching functions. IEEE Trans Magnet. 2009;45(10):4672–5.

[16] Sant A, Rajagopal KR, Sheth NK. Permanent magnet synchronous motor drive using hybrid PI speed controller with inherent and non-inherent switching functions. IEEE Trans Magnet. 2011;47(10):4088–91. doi: insert-doi-here

[17] Zerikat M, Chekroun S. Design and implementation of a hybrid fuzzy controller for a high-performance induction motor. In Proceeding of World Academy of Science, Engineering and Technology. Int J Comput Inf Eng. 2007;20:263–9.

[18] Rubaai A, Ricketts D, Kankam MD. Experimental verification of a hybrid fuzzy control strategy for a high-performance brushless DC drive system. IEEE Trans on Ind Applicat. 2001;37(2):503–12. doi: 10.1109/28.913715

[19] Singh B, Singh BP, Dwivedi S. DSP based implementation of hybrid fuzzy PI speed controller for direct torque controlled permanent magnet synchronous motor drive. International J Emerg Elect Power Syst. 2007;8(2):1–22. doi: 10.2202/1553-779x.1246

[20] Nakao N, Akatsu K. Suppressing pulsating torques: torque ripple control for synchronous motors. IEEE Ind Appl Mag. 2014;20(6):33–44. doi: 10.1109/mias.2013.2288383

[21] Truong P, Flieller D, Nguyen NK, Mercklé J, Sturtzer G. Torque ripple minimization in non-sinusoidal synchronous reluctance motors based on artificial neural networks. Elect Power Syst Res. 2016;140:37–45.

[22] Flieller DN, Nguyen K, Wira P, Sturtzer G, Abdeslam D, Merckle J. A self-learning solution for torque ripple reduction for nonsinusoidal permanent-magnet motor drives based on artificial neural networks. IEEE Trans Ind Electron. 2014;61(2):655–66. doi: 10.1109/TIE.2013.2281234

[23] Feng G, Lai C, Kar NC. A closed-loop fuzzy-logic-based current controller for pmsm torque ripple minimization using the magnitude of speed harmonic as the feedback control signal. IEEE Trans Ind Electron. 2017;64(4):2642–53. doi: 10.1109/tie.2016.2631524

[24] Xiao G, Wang X, Tu W, Tang L, Zhang H, Yang K. A sensor less control of surface-mount permanent magnet synchronous motor based on rotor flux estimation. 20th International Conference on Electrical Machines and Systems (ICEMS). Sydney, NSW: 2017. p. 1–4.

[25] Devanshu A, Singh M, Kumar N. An improved nonlinear flux observer based sensorless FOC IM drive with adaptive predictive current control. IEEE Trans Power Electron. 2020;35(1):652–66.

[26] Devanshu A, Singh M, Kumar N. Nonlinear flux observer-based feedback linearisation control of IM drives with ANN speed and flux controller. Int J Electron. 2021;108(1):139–61.

[27] Kamel HM, Hasanien HM, Ibrahim HE. Speed control of permanent magnet synchronous motor using fuzzy logic controller. Proceedings of the IEEE International Conference on Electric Machines and Drives. Miami, Florida, USA.: 2009. p. 1587–91.

[28] Chaoui H, Sicard P. Adaptive Fuzzy Logic Control of Permanent Magnet Synchronous Machines With Nonlinear Friction. IEEE Trans Ind Electron. 2012;59(2):1123–33. doi: 10.1109/tie.2011.2148678

[29] Yu JS, Kim SH, Lee BK, Won CY, Hur J. Fuzzy-logic-based vector control scheme for permanent-magnet synchronous motors in elevator drive applications. IEEE Trans Ind Electron. 2007;54(4):2190–200. doi: 10.1109/tie.2007.894692

[30] Krishnan R, Beutler AJ. Performance and design of an axial field permanent magnet synchronous motor servo drive. Proceedings of IEEE IAS Annual Meeting. 1985. p. 634–40.

[31] Lajoie-Mazenc M, Villanueva C, Hector J. Study and implementation of hysteresis controlled inverter on a permanent magnet synchronous machine. IEEE Trans on Ind Applicat. 1985;IA-21(2):408–13. doi: 10.1109/tia.1985.349662

[32] Aner M, Benaifa E, Nowicki A. A PMSM drive design with inverter-stage soft-switching hysteresis current control and space vector modulation for two-level operation of a very sparse matrix converter. IEEE Electrical Power & Energy Conference (EPEC). Montreal, QC; 2009. p. 1–8.

[33] Suryakant M, Sreejeth M, Singh M. Performance Analysis of PMSM Drive using Hysteresis Current Controller and PWM Current Controller. Proceedings of the 2018 IEEE International Students’ Conference on Electrical, Electronics and Computer Science (SCEECS). Bhopal, India; 2018. p. 1–5.

[34] Suryakant M, Sreejeth M, Singh M. Sensor-less control of PMSM drive with BEMF based MRAC algorithm. Proceedings of the 2019 International Symposium on Advanced Electrical and Communication Technologies (ISAECT). Rome, Italy; 2019. p. 1–6.

[35] Panda SK, Xu J-X, Qian W. Review of torque ripple minimization in PM synchronous motor drives. 2008 IEEE Power and Energy Society General Meeting - Conversion and Delivery of Electrical Energy in the 21st Century. Pittsburgh, PA; 2008. p. 1–6.

[36] Shukla S, Sreejeth M, Singh M. Minimization of ripples in stator current and torque of PMSM drive using advanced predictive current controller based on deadbeat control theory. J Power Electron. 2021;21:142–52.

